# Periodontal grading—estimation of responsiveness to therapy and progression of disease

**DOI:** 10.1007/s00784-024-05678-3

**Published:** 2024-05-01

**Authors:** Caspar Victor Bumm, Christina Ern, Julia Folwaczny, Uta Christine Wölfle, Katrin Heck, Nils Werner, Matthias Folwaczny

**Affiliations:** 1 Department of Conservative Dentistry and Periodontology, University Hospital, LMU Munich Goethestraße 70, 80336 Munich, Germany; 2 Private Practice, Munich, Germany

**Keywords:** Periodontal diseases, Periodontitis, Classification, Disease progression

## Abstract

**Objective:**

To investigate the capability of periodontal grading to estimate the progression of periodontal disease and the responsiveness to therapy.

**Materials and methods:**

Eighty-four patients who underwent non-surgical therapy (NST) were included. Direct and indirect evidence of progression were determined according to the current classification. Responsiveness to therapy was examined using mean pocket probing depths reduction (PPDRed), reduction of bleeding on probing (BOPRed), and the rate of pocket closure (%PC) after six months.

**Results:**

Statistical analysis revealed no agreement between direct and indirect evidence in grading periodontitis (κ = 0.070). The actual rate of progression as determined by longitudinal data was underestimated in 13% (*n* = 11), overestimated in 51% (*n* = 43) and correctly estimated in 30% (*n* = 36) by indirect evidence. No significant differences in responsiveness to therapy were observed in patients graded according to direct evidence. Using indirect evidence, patients assigned grade C showed more PPDRed but less BOPRed and lower %PC compared to grade B.

**Conclusion:**

The present data indicate that indirect evidence may lead to inaccuracies compared to direct evidence regarding the estimation of periodontal progression. However, indirect evidence seems to be more suitable in the estimation of responsiveness to therapy than direct evidence, helping to identify cases that are more likely to require additional therapies such as re-instrumentation or periodontal surgery.

**Clinical relevance:**

Regarding the estimation of disease progression and responsiveness to periodontal therapy, accuracy and reliability of both direct and indirect evidence are limited when grading periodontitis.

## Introduction

The current classification of periodontal and peri-implant diseases and conditions published in 2018 [1–3] features a variety of differences from the previous classification system [4]. The most important novelty is the introduction of a severity-based staging and grading system as common in other subfields of medicine. Whereas the 1999 classification distinguished between different types of periodontitis (i.e., chronic/aggressive) and assumed different entities, the new classification considers the scientific results of the last decades, which eventually have not provided evidence of a different aetiology or pathogenesis of the different forms of periodontitis. The new classification further includes a system that maps different degrees of severity and complexity, as well as predictive factors that play a role in the individual prognosis of periodontitis and the assessment of risk profiles [3, 5].

While staging describes the current severity and complexity of the individual disease, grading aims to predict the future progression of the disease and to estimate the responsiveness to standard therapy. Periodontal grading can be determined using either longitudinal data in form of older quality radiographs (i.e., direct evidence of progression) or the relation between maximum radiographic bone loss (RBL) in percent and patient age (i.e., indirect evidence of progression) [3].

Independently of the primary criterion (direct/indirect evidence) used to determine the progression rate, so-called grade modifiers that indicate higher “disease progression or less responsiveness to bacterial reduction” [3] may shift the grade score to a higher level. Currently acknowledged grade modifiers are the two established risk factors of periodontitis, smoking and diabetes mellitus [6–9] depending on their degree of severity. Besides these risk factors, Tonetti et al. emphasize the use of other available information [3] including the case phenotype, specific biomarkers or the risk of a systemic effect of periodontitis [3]. On case phenotype (describing the amount of biofilm deposits in relation to periodontal destruction) as part of indirect evidence that should aid to identify patients with high progression rates the classification remains relatively vague. Since so far exact quantifiable methods are not mentioned comparability and eventually usability of this parameter can be considered as low. Regarding biomarkers, although a plethora related to periodontitis has been investigated in the past, at present there is no sufficient evidence for the use of a single biomarker that may accurately predict periodontal progression [10–13].

Therefore, to date, grading a periodontitis case is most likely limited to longitudinal radiographic data or indirect evidence in terms of the % RBL/age index and the two accepted grade modifiers smoking and diabetes mellitus. Whether grading in this manner can meet the goals proposed by Tonetti et al., i.e. estimation of disease progression and future responsiveness to standard therapy, however, is unclear.

The aims of this diagnostic accuracy study were therefore 1.) to compare direct and indirect evidence to analyse their consistency in estimating the progression of disease and 2.) to investigate the correlation of grading (direct/indirect) on the responsiveness to non-surgical therapy (NST).

## Methods

This retrospective study was approved by the Ethics Committee of the Medical Faculty of the Ludwigs-Maximilians-University, Munich (No. 22–0669) and was conducted in accordance with the principles of good clinical practice and the Declaration of Helsinki, as revised in 2013. The study is registered at the German Clinical Trials Register (DRKS00028923).

### Study population

Out of 759 patients who had undergone non-surgical therapy (NST) between February 2011 and March 2016 in the undergraduate program at the Department of Conservative Dentistry and Periodontology, University Hospital, LMU Munich 84 patients were included in the study [14]. All patients met the following inclusion criteria: 1. Age ≥ 18 years, 2. Diagnosis of periodontitis according to the current classification [15] 3. A Periodontal chart with documentation of pocket probing depths and bleeding on probing at six sites/tooth, before NST 4. Evaluable panoramic radiographs at the beginning of step 1 of periodontal therapy and at least five years prior, 5. A complete history of diabetes and self-reported history of smoking. The exclusion criteria were 1. Periodontal treatment at the Department of Conservative Dentistry and Periodontology and self-reported history of periodontal treatment alio loco within 5 years preceding baseline, 2. Non-comparability or absence of two panoramic radiographs in the study period, 3. Pregnancy, 4. Unavailable haemoglobin A1c values (HbA1c) in patients diagnosed with diabetes, 5. Smokers not reporting the number of cigarettes/day and 6. Adjunctive systemically or locally administered antibiotics during NST. 

### Clinical parameters

Periodontal charts were conducted at baseline and at the time of re-evaluation (six months after NST) by two experienced periodontists (CE and RH, inter-rater reliability in periodontal probing, Cohen’s kappa: 0.82) [16] containing pocket probing depths (PPD) and bleeding on probing (BOP) at six sites per tooth. PPD was measured to the nearest mm using a PCP-12 periodontal probe (Hu-Friedy, Chicago, USA) with a trained probing force of approximately 0.2–0.3 N as proposed by Gabathuler and Hassell [17]. BOP was assigned 30 s after probing according to van der Weijden et al. [18]. Periodontal pockets at baseline were defined as PPD > 3 mm. Persisting pockets at re-evaluation were defined as PPD = 4 mm with BOP or > 4 mm [2].

### Periodontal treatment

Periodontal treatment was performed by undergraduate students supervised by the aforementioned operators (CE, RH). Prior to non-surgical therapy, all patients were subjected to the first step of therapy as proposed by Sanz et al. [19], including detailed information about the aetiology, pathogenesis, risk factors and treatment of periodontitis and received oral hygiene instructions as well as professional mechanical plaque removal (PMPR). Subgingival debridement was performed under local anesthesia at all teeth with PPD > 3 mm using sonic devices (SONICflex^TM^, Kavo, Biberach, Germany) and a standard set of Gracey curettes (SG5/6, SG7/8, SG 13/14, SG15/16, Hu-Friedy, Chicago, USA), without restrictions in terms of time [20].

### Radiographic analysis

Panoramic radiographs were taken using digital X-ray technology and analyzed with the corresponding software (Sidexis XG 2.63, Dentsply Sirona Deutschland GmbH, Bensheim, Germany). The following landmarks as described by Nibali et al. [21] were established: cemento-enamel junction (CEJ), radiographic apex and bottom of the alveolar bone crest (bone level, BL). Measurements of the distances between CEJ and apex (CEJ-apex) and CEJ and BL (CEJ-BL) were performed at the most severely affected tooth to the nearest millimetre (mm) calculating the percentage RBL by dividing CEJ-BL/CEJ-apex [21, 22]. In case of absent CEJ due to prosthodontic restorations, the restoration margin was defined as an alternative landmark to CEJ.

All radiographs were examined by the same two raters each (CVB, NW). In advance, a period of examiner alignment in terms of a training exercise was conducted using 20 panoramic radiographs not included in the study. Further, intra-rater reliability was determined by repeated measurement of 20 randomly assigned radiographs of the study cohort. In the event of > 10% deviation of the RBL measurements between the two raters, radiographs were re-examined by both examiners until consensus was reached.

### Grading

Grading was performed using direct and indirect evidence resembling the primary grading criteria. For direct evidence, the progression of RBL was measured by comparing RBL in mm at least five years prior to baseline and at baseline. Grading was assigned accordingly (Grad A: no progression of RBL, Grade B: < 2 mm progression of RBL, Grade C: ≥ 2 mm progression of RBL) [23]. For indirect evidence, the percentage RBL was calculated as described above and divided by the patient’s age at baseline and five years prior to baseline resulting in quotients that are expressed as a decimal number. Grading was assigned accordingly (Grade A: < 0.25, Grade B: 0.25–1.0, Grade C: > 1.0). Both methods were applied to the most severely affected tooth, however, not considering bone loss around third molars or at the distal aspects of second molars.

### Sample size

Sample size calculation was done based on previous data as reported by Winkler et al. [24] under the assumption of an expected kappa value of 0.4, a minimum acceptable kappa of 0.7 and a proportion of outcome (i.e. periodontal grade C according to radiographic bone loss) indicating a minimum sample size of 66 individuals to reach a power (1-β) of 0.9.

### Statistical analysis

All analyses were performed using the SPSS Statistic software (Version 26.0; IBM, Corp., New York, USA). The normality of data was tested using Shapiro–Wilk-Test. Differences between patients with Grades A, B and C were compared using an Analysis of Variance (ANOVA) for continuous variables, the Kruskal–Wallis Test for ordinal and skewed variables, and the Chi-square test for nominal variables. Post hoc pairwise analysis for Kruskal–Wallis Test was done using Dunn-Bonferroni-Test. Intra- and inter-rater reliability were assessed using the Pearson correlation coefficient (Pearson's r). Differences between the grading systems were assessed using Cohen’s kappa (κ). The two-sided significance level was set at α = 0.05 for all tests.

## Results

### Patient characteristics

**Fig. 1:**
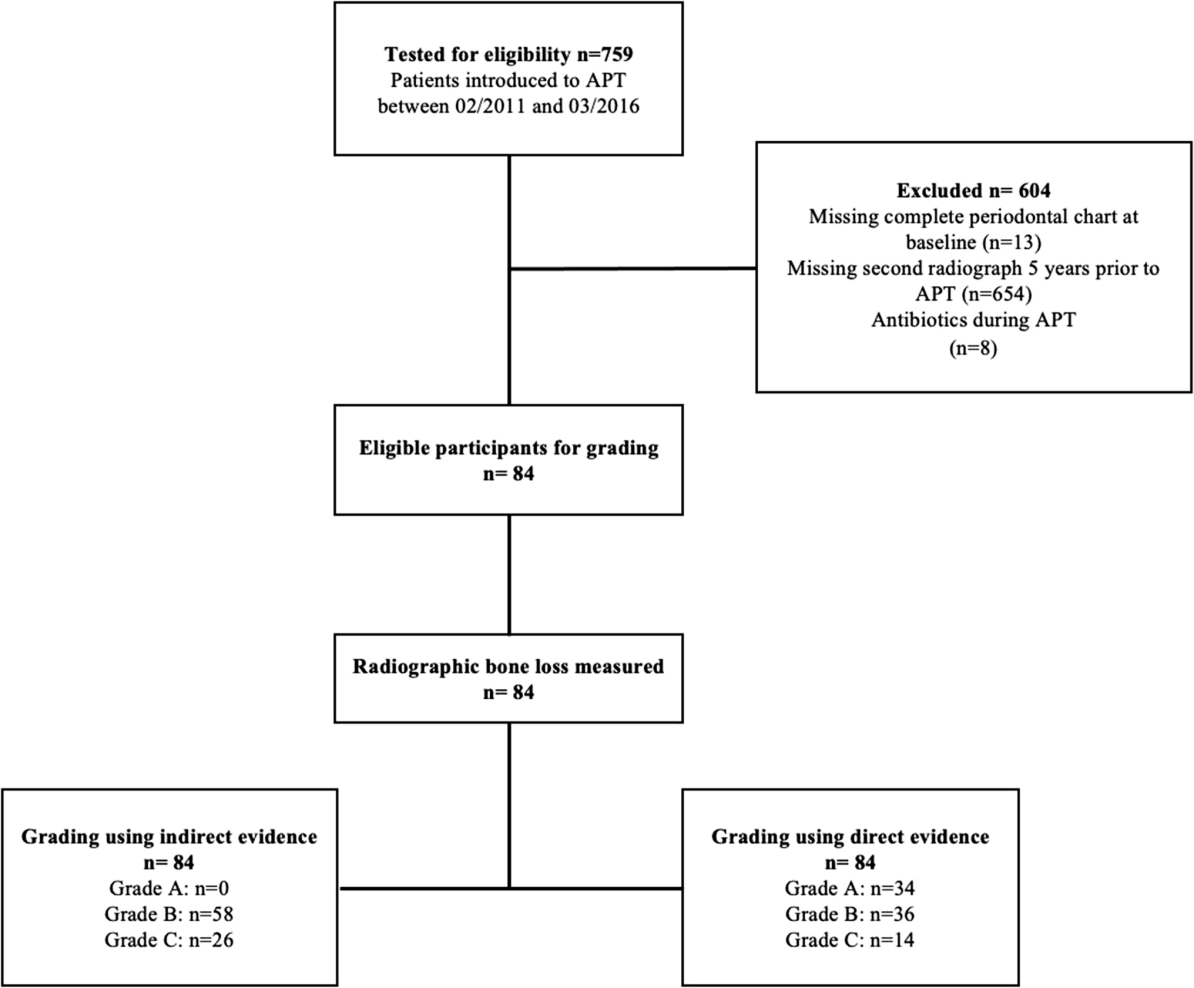
Flow diagram of the process of trial inclusion/exclusion; APT, active periodontal therapy

Seven hundred fifty-nine patients received NST between February 2011 and March 2016. The final analysis included 84 Patients who met the inclusion criteria described above and were retrospectively graded at baseline via direct and indirect evidence of progression (Fig. 1).

Patients’ mean age at baseline was 63 (± 11) years, and the male-to-female ratio was 45%/55%. Eighty-three patients were non-smokers, one patient was a smoker (≥ 10 cigarettes/d) (Table 1). Eight patients were excluded due to the application of systemic or local antibiotics during NST in order to dismiss potential bias regarding therapy outcomes.

**Table 1:** Patient characteristics

Variable	*n*
Patients (total)	84 (100)
Age	63 ± 11
Female	45 (54)
Smokers (total)	1 (1)
Smokers (< 10 cigarettes/d)	0 (0)
Smokers (≥ 10 cigarettes/d)	1 (1)
PI, %	36 ± 22
BOP, %	30 ± 17
Teeth with mobility, %	17 ± 23

Results are provided as mean ± standard deviation or frequencies (%). BOP, bleeding on probing; PI, plaque index

### Comparison of direct and indirect evidence at baseline

Grading was applied using direct and indirect evidence at baseline and five years prior to baseline. As depicted in Table 2, direct evidence revealed that 34 (41%) patients had a low rate of progression (Grade A), 36 (42%) showed a moderate rate (Grade B) and 14 (17%) had a high rate (Grade C). At baseline indirect evidence suggested that zero patients had a low, 58 patients a moderate and 26 had a high rate of progression (Table 2). Comparison of the grades assessed either by direct or indirect evidence using Cohen’s kappa showed no agreement between the two methods (κ = 0.070) [25].

**Table 2:** Comparison of direct and indirect evidence in grading. A: Direct evidence (baseline) versus indirect evidence at baseline, B: Direct evidence (baseline) versus indirect evidence five years prior

A		Direct evidence (baseline)			
		Grade A	Grade B	Grade C	*n* (%)
Indirect evidence (baseline)	Grade A	0	0	0	0
	Grade B	27 (79)	25 (69)	6 (43)	58 (69)
	Grade C	7 (21)	11 (31)	8 (57)	26 (31)
	*n* (%)	34 (41)	36 (43)	14 (17)	84 (100)
B		Direct evidence (baseline)			
		Grade A	Grade B	Grade C	*n* (%)
Indirect evidence (5 years prior)	Grade A	0	0	1(7)	1
	Grade B	19 (56)	27 (75)	10 (18)	56 (67)
	Grade C	15 (44)	9 (25)	3 (21)	27 (31)
	*n* (%)	34 (41)	36 (43)	14 (17)	84 (100)

Results are presented as total numbers and frequencies (%). Direct evidence was assessed using longitudinal radiographic data, comparing RBL five years prior to APT with RBL at baseline. Indirect evidence was assessed using RBL/age at baseline (A) and five years prior (B)

*n*, number of patients, RBL, radiographic bone loss; APT, active periodontal therapy

### Comparison of direct and indirect evidence five years prior to NST

Five years prior to baseline indirect evidence resulted in one patient with a low, 56 with a moderate and 27 with a high rate of progression. Comparing this to the actual disease progression in the form of direct evidence, there was an overrating of 51% (*n* = 43), a correct estimation of 36% (*n* = 30), and an underrating of 13% (*n* = 11) of all patients and no agreement between predicted and actual progression (κ = 0.020).

### Intra- and inter-rater reliability

Intra-rater reliability was strong for both examiners (CVB = 0.94, NW = 0.92; *p* < 0.001). Likewise, Pearson's r revealed a significant linear correlation comparing the two examiners (*r* = 0.96; *p* < 0.001).

### Periodontal characteristics and estimation of responsiveness to therapy

Clinical parameters obtained at baseline and re-evaluation (Table 3) were correlated with the grades assigned either based on direct or indirect evidence. To evaluate the responsiveness to NST pocket probing depths reduction (PPDRed), the rate of pocket closure (%PC) and reduction of bleeding on probing (BOPRed) were calculated (Table 3).

**Table 3:** Therapy related parameters alongside non-surgical treatment

	PPD_BASE	PPD_REV	PPD_RED	BOP_BASE	BOP_REV	BOP_RED	PC
ALL	4.87 ± 1.16	4.07 ± 1.64	0.79 ± 1.4	30 ± 17	24 ± 14	7 ± 16	45 ± 31
Indirect evidence							
Grade A							
Grade B	4.76 ± 1.06	4.16 ± 1.69	0.59 ± 1.33	29 ± 15	23 ± 13	6 ± 17	47 ± 29
Grade C	5.02 ± 1.23	3.95 ± 1.55	1.07 ± 1.48	33 ± 22	25 ± 15	9 ± 15	39 ± 36
*p*-value	** < 0.001**	**0.045**	** < 0.001**	0.503	0.772	0.504	0.391
Direct evidence							
Grade A	4.80 ± 1.14	4.09 ± 1.70	0.70 ± 1.41	30 ± 17	24 ± 15	7 ± 15	48 ± 32
Grade B	4.89 ± 1.07	4.10 ± 1.54	0.79 ± 1.31	32 ± 18	24 ± 12	8 ± 19	41 ± 24
Grade C	5.13 ± 1.55	3.85 ± 1.76	1.27 ± 1.77	23 ± 14	23 ± 15	2 ± 15	47 ± 48
*p*-value	**0.045**^**a**^	0.409	**0.002**^**ac**^	0.446	0.980	0.723	0.715

Results are provided as mean ± standard deviation. PPD values are presented in millimeters, BOP and PC values are presented as frequencies (%). Grading is assessed through direct evidence of progression. BASE, baseline; PC, pocket closure; PPD, probing depth of all pockets > 3 mm; REV, re-evaluation; RED, reduction

^a^Grade A vs. Grade C < 0.05

^b^Grade A vs. Grade B < 0.05

^c^Grade B vs. Grade C < 0.05

Bold indicates statistically significant values (*P* < 0.05)

The mean PPD at baseline was 4.87 ± 1.16 mm and differed significantly between groups. The highest PPD at baseline were found in patients assigned Grade C using both direct (5.13 ± 1.55 mm) and indirect (5.02 ± 1.23 mm) evidence. Likewise, a significantly increased reduction of PPD could be observed in these groups at re-evaluation (direct evidence: 1.27 ± 1.77 mm, indirect evidence: 1.07 ± 1.48 mm).

The mean BOP at baseline was 30 ± 17% and 24 ± 14% at re-evaluation, the difference between groups did not reach significance. However, patients assigned Grade C using indirect evidence showed higher initial BOP than all other groups (33 ± 22%).

The mean rate of pocket closure was 45 ± 31%, showing the minimum PC rate of 39 ± 36% among patients assigned Grade C using indirect evidence.

When dividing the cohort by the mode of grading (direct/indirect) the results for PPDRed, %PC and BOPRed did not differ significantly in the groups graded by direct evidence (Table 3). Grading assigned by indirect evidence, on the contrary, revealed increased PPDRed, but less %PC and BOPRed in subjects assigned to Grade C as compared to Grade B (Table 3).

## Discussion

The objective of grading is to identify cases of periodontitis “progressing at a greater rate than is typical for the majority of the population or responding less predictably to standard therapy” [23]. In this context, the current classification has defined two major goals of grading. Apart from the estimation of future risk for disease progression and responsiveness to standard therapy as well as the guidance of intensity of therapy and monitoring, grading tries to estimate the potential impact of periodontitis on general health.

Since previous radiographs and/or periodontal findings are mostly unavailable at diagnosis indirect evidence in terms of the % RBL/age index is commonly used for grading periodontitis. To investigate whether grading using indirect evidence alone can provide a reliable prognosis of the future course of periodontitis, the present study evaluated the consistency between direct and indirect evidence regarding the estimation of disease progression and responsiveness to therapy. Herein clinical and radiographic findings at the time and at least five years prior to NST of a patient cohort referred to the Department of Conservative Dentistry and Periodontology were analysed.

This retrospective trial revealed partially inconsistent results when comparing direct and indirect evidence as used for periodontal grading at both time points considered herein. Most importantly, the indirect method was neither able to estimate the past nor the future progression of disease correctly in the present cohort. Despite its widespread use, many authors have emphasized the limited scientific evidence on the predictive value of the % RBL/age index [26, 27]. Lang and Tonetti incorporated this parameter in their widely used periodontal risk assessment (PRA) published in 2003 bearing in mind that the cumulative nature of periodontal destruction may lead to overestimation or underestimation of true progression [28, 29]. In cases of advanced isolated defects and lesions with local etiological factors, for instance, an individual’s progression rate is likely to be overestimated using the index. While in generalized advanced cases, progression rates might be underestimated [27]. Presumably, these restrictions are partially reflected by the current results, since with only one smoker and no patients with diabetes mellitus grading was based predominantly on the index without grade modifiers.

Regarding the estimation of responsiveness to therapy, independently of the primary criterion (direct/indirect) patients assigned grade C showed significantly higher pocket probing depths at baseline and significantly higher improvement in terms of PPD reduction after six months.

While grading determined by direct evidence showed no correlation to BOP reduction or %PC, indirect evidence revealed significantly less BOP reduction in patients with grade C. These patients also showed %PC clearly below the average recently described in a systematic review [30], leading to the assumption that indirect evidence might be more accurate in the estimation of responsiveness to therapy than direct evidence. It is notable, that overall %PC found in this cohort were inferior to those recently published by Suvan et al. [31] as well as Citterio and co-workers [30]. It must be mentioned, however, that the studies included in these reviews either did not directly report on PC or used different definitions of PC (e.g. ≤ 3 mm, ≤ 4 mm, ≤ 5 mm). In this study the rather strict definition of PC proposed by the current classification (≤ 3 mm or = 4 mm without BOP) was used. The appearance of a sub-standard response to therapy in this cohort might therefore be at least partially explained by divergent definitions of “pocket closure” present in the literature.

The seemingly contradictory results in grade C patients (higher PPDRed but lower %PC) might be due to the fact that patients assigned grade C had initially deeper mean PPD and also a higher frequency of deep PPD (≥ 6 mm) than those in grade B at baseline. Here the well-known fact, that deeper PPD respond more favorably to NST in terms of PPDRed becomes evident [20, 31]. On the other hand, even though PPDRed was better for this sub-cohort, the relative amount of pockets closed and the amount of BOP reduction was worse compared to grade B. Thus, in our cohort, the need for additional therapy such as re-instrumentation or surgical therapy was higher in patients graded C according to indirect evidence than in those graded B.

Whether these clinical findings actually support the capability of indirect evidence to estimate the responsiveness to therapy on a patient-based level, however, remains questionable. Presumably, the findings rather show that initially more complex cases (e.g. PPD ≥ 6 mm) with more progressive tissue defects tend to leave more residual pockets than cases with less complexity. Thus, resulting in an increased need for additional therapy.

The results of this study critically address the objectives of periodontal grading using the methods available at present. Regarding the above-mentioned objectives, grading should be considered as a patient-related category of periodontal diagnosis. Since it does not aim to identify teeth or sites, but individuals that show a higher progression rate than usual and respond less favorably to therapy. In contrast, staging uses different tooth- and site-related factors to categorize the severity and complexity of the disease, such as bone loss, PPD, furcation involvement, etc. and can be considered as a tooth or site-level category of diagnosis.

To date, however, since no reliable biomarkers or other resilient indicators may help to meet the goals of grading, grading itself is also based mainly on tooth-related factors, e.g., bone loss. When indirect evidence is used, bone loss at the most severely affected tooth is described as a function of age, adding a parameter on the patient-level. In the present cohort, the latter seemed to have a positive effect on the estimation of responsiveness to cause-related therapy but not on the progression of the disease. Clearly, the results of this study support the need for further research on patient-related factors truly identifying individuals responding less favourable to therapy and presenting with higher progression rates than usual.

The following limitations of this study need to be addressed. Periodontal treatment was delivered by undergraduate students, whereas in most of the studies included in the reviews by Citterio et al. [30] and Suvan et al. [31] periodontists or dental hygienists performed NST, thus mitigating comparability of the data. Considering varying levels of skill and experience in general dentists and hygienists, however, our setting might be related more closely to everyday clinical practice [32–34] contributing to the external validity of the results, eventually.

Regarding patient characteristics of the study cohort however, generalizability is limited, since it comprised merely one smoker and no patients diagnosed with diabetes, thus not considering two well-accepted risk factors for periodontal progression.

Although patients having received periodontal treatment in the Department of Conservative Dentistry and Periodontology and those with a self-reported history of periodontal treatment within 5 years preceding baseline were excluded from the analysis, it was not possible to conclusively rule out, whether any periodontal has been performed elsewhere. It might be assumed, that some patients have at least received components of step 1 therapy, e.g. professional mechanical plaque removal during check-up appointments alio loco, thus representing a limitation of the results regarding the ability of direct and indirect evidence to properly estimate disease progression.

Both direct and indirect evidence were applied to the tooth most severely affected by periodontal destruction. This might have led to an underestimation of the true progression rates, regarding the whole dentition. On the other hand, the % RBL/age index should reflect the progression of periodontal destruction of the entire dentition [27], which is why we focused on the index tooth. Although periapical radiographs are standard for detecting periodontal bone loss, panoramic radiographs are widely used among dentists for initial diagnosis, which is why we used them in this study [26, 35]. Clinical attachment levels (CAL) were not continuously collected in this study and are consequently not reported. Although CAL is an important parameter in staging periodontitis and to assess progression over time clinically, according to the current classification the gold standard to determine the progression rate is longitudinal data in form of older diagnostic quality radiographs as used in this study. Finally, other factors that were not recorded might have had a significant impact on progression rates and therapy response at baseline.

The present data indicate that indirect evidence may lead to inaccuracies compared to direct evidence regarding the estimation of periodontal progression. Over- and underestimation of actual progression rates most likely occur due to limitations inherent in indirect evidence regarding the cumulative nature of periodontal attachment loss. However, indirect evidence seems to be more suitable in the estimation of responsiveness to therapy and consequently in identifying cases that are more likely to require additional therapies such as re-instrumentation or periodontal surgery than direct evidence. Whether the incorporation of risk factors, biological or genetic data might overcome these inaccuracies as well as the potential impact on cost-effectiveness for healthcare providers regarding monitoring intervals based on grading warrants further investigation.

## Data Availability

The data that support the findings of this study are available from the corresponding author upon reasonable request.
